# Validation of the Romanian Version of the Halitosis Associated Life-Quality Test (HALT) in a Cross-Sectional Study among Young Adults

**DOI:** 10.3390/healthcare11192660

**Published:** 2023-09-30

**Authors:** Raluca Briceag, Aureliana Caraiane, Gheorghe Raftu, Melania Lavinia Bratu, Roxana Buzatu, Liana Dehelean, Mariana Bondrescu, Felix Bratosin, Bogdan Andrei Bumbu

**Affiliations:** 1 Faculty of Dental Medicine, Ovidius University of Constanta, 7 Ilarie Voronca Street, 900684 Constanta, Romania; raluca.briceag@365.univ-ovidius.ro (R.B.); gheorgheraftu@yahoo.com (G.R.); 2 Department of Oral Rehabilitation, Faculty of Dental Medicine, Ovidius University of Constanta, 900684 Constanta, Romania; drcaraiane@yahoo.com; 3 Department of Psychology, Faculty of General Medicine, “Victor Babes” University of Medicine and Pharmacy Timisoara, Eftimie Murgu Square 2, 300041 Timisoara, Romania; bratu.lavinia@umft.ro; 4 Department of Dental Aesthetics, Faculty of Dental Medicine, “Victor Babes” University of Medicine and Pharmacy Timisoara, Revolutiei Boulevard 9, 300041 Timisoara, Romania; 5 Department of Neurosciences and Psychiatry, Faculty of General Medicine, “Victor Babes” University of Medicine and Pharmacy Timisoara, Eftimie Murgu Square 2, 300041 Timisoara, Romania; lianadeh@umft.ro (L.D.); mariana.bondescu@umft.ro (M.B.); 6 Department of Infectious Diseases, Faculty of General Medicine, “Victor Babes” University of Medicine and Pharmacy Timisoara, Eftimie Murgu Square 2, 300041 Timisoara, Romania; felix.bratosin@umft.ro; 7 Department of Dental Medicine, Faculty of Medicine and Pharmacy, University of Oradea, 410073 Oradea, Romania; bogdanbumbu@uoradea.ro

**Keywords:** halitosis, quality of life, oral health

## Abstract

Halitosis is a common condition with a significant impact on individuals’ quality of life. The Halitosis Associated Life-Quality Test (HALT) is a reliable instrument for measuring this impact. This study aimed to introduce and validate the Romanian translation of the HALT questionnaire (R-HALT). We hypothesized that the R-HALT would demonstrate good reliability and validity in measuring the impact of halitosis on quality of life among Romanian teenagers and young adults. Our objectives were to translate and adapt the HALT, validate it among a cross-sectional group, and evaluate the extent of halitosis in this population. A multicentric cross-sectional design followed, which was approved by the Ethics Research Committee in Romania. The translation process involved independent translations, retro-translations, expert review, and pre-testing. The psychometric properties were evaluated among 150 patients (mean age 23.6 ± 1.8; 51% males) at dental clinics, including reliability, convergent, and discriminant validity, using accepted statistical measures such as Cronbach’s alpha and Intraclass Correlation Coefficient (ICC). The R-HALT revealed strong internal consistency with Cronbach’s alpha values ranging from 0.93 to 0.96, and an ICC value of 0.87 (95% CI = 0.70–0.99), demonstrating excellent test–retest reliability. Mean scores of individual items ranged from 0.82 ± 0.94 (Q3) to 3.23 ± 1.15 (Q11). The corrected item–total correlation ranged from 0.30 (Q2) to 0.90 (Q19). Organoleptic test scores diagnosed 41% (62 patients) with a score of 2, with increasing severity in 9% (13 patients) with a score of 5. The R-HALT exhibited robust reliability and validity in assessing the impact of halitosis among Romanian teenagers and young adults. The questionnaire is a strong tool for understanding, diagnosing, and managing halitosis in Romania, ultimately aiming to improve the quality of life of affected individuals. Further studies with diverse populations could enhance the applicability of the R-HALT.

## 1. Introduction

Halitosis, commonly referred to as bad breath, is a prevalent health concern that has potential implications for the psychological well-being and social interactions of affected individuals [1,2]. It is estimated that approximately 30–50% of the global population suffers from this condition, with varying degrees of severity [3,4]. Due to its significant social impact and potential indicators of underlying health issues, halitosis is increasingly recognized as a public health matter that merits thorough investigation [5,6].

The diagnosis and management of halitosis often pose considerable challenges, in part due to the subjective nature of the condition [7]. A person’s self-perception of their breath may not correspond with objective assessments, leading to conditions such as genuine halitosis, pseudohalitosis, and halitophobia [8,9]. Furthermore, various factors can influence the presence and severity of halitosis, including physiological conditions, dietary habits, oral hygiene practices, and certain systemic diseases [10]. Despite these complexities, the highest prevalence of halitosis originates from the oral cavity in approximately 85% of cases [11], making dental professionals central to its detection and management.

To effectively assess and monitor halitosis, it is crucial to have valid, reliable, and easily applicable tools. One such instrument is the Halitosis Associated Life-Quality Test (HALT), a questionnaire developed to measure the impact of halitosis on various aspects of an individual’s life, such as social interactions, psychological state, and overall quality of life [12]. By allowing the assessment of halitosis from the patient’s perspective, HALT aids in the detection of halitosis cases and the subsequent evaluation of treatment efficacy.

In Romania, a detailed understanding of halitosis, particularly among the youth, is somewhat limited, although recent studies have begun to shed light on their oral hygiene knowledge and habits. For instance, while a notable percentage of Romanian students are aware of proper toothbrushing techniques and the importance of plaque removal, they exhibit limited knowledge about the remineralization capabilities of brushing [13]. Moreover, their oral hygiene practices vary considerably, with differing attitudes observed based on their level of education [14]. Against this backdrop, the introduction and validation of the Romanian version of the Halitosis Associated Life-Quality Test (HALT) is timely, aiming to bridge the knowledge gap and enhance the understanding and management of halitosis in the Romanian young adult and teenage population.

Despite the recognized utility of HALT, its application has been limited in certain regions due to language barriers [15,16]. For example, in Romania, where halitosis is a common problem in the adult populations [17,18], there is currently no validated Romanian version of HALT. Also, the statistics discussing the prevalence of halitosis among the Romanian youth are insufficient or non-existent, leaving a knowledge gap. Moreover, the lack of appropriate tools hampers the detection, management, and understanding of halitosis in this particular group.

In light of this gap in the literature, this study aims to introduce and validate the Romanian translation of the HALT questionnaire. We hypothesize that the Romanian version of the HALT questionnaire will demonstrate good reliability and validity in measuring the impact of halitosis on quality of life among Romanian teenagers and young adults. The study objectives are to translate and adapt the HALT questionnaire into Romanian; to validate the Romanian version of HALT among a cross-sectional group of teenagers and young adults; and thirdly, to evaluate the extent of halitosis and its impact on quality of life in this population. By accomplishing these objectives, we aim to provide a robust, applicable tool for better understanding, diagnosing, and managing halitosis in Romania, ultimately improving the quality of life of affected individuals.

## 2. Materials and Methods

### 2.1. HALT Overview

The Halitosis Associated Life-Quality Test (HALT) was conceived originally in English by Kizhner et al. [19] to measure the impact of halitosis on quality of life, demonstrating reliable psychometric characteristics. HALT is a specialized tool tailored for the exhaustive assessment of the physical, social, and psychological implications of halitosis in adults. HALT consists of 20 items, each scored on a 5-point scale that ranges from 0 to 5, where 5 is the most negative answer. The total score is obtained by adding the individual scores from the 20 items, providing a range from 0 to 100. Higher scores reflect a more substantial impact of halitosis on the quality of life related to oral health.

### 2.2. Study Design and Ethics

The current study followed a multicentric cross-sectional design. This study’s protocol received approval from the Ethics Research Committee (E-787, from 8 February 2023) and was developed by a multicentric, inter-disciplinary team from the “Ovidius” University of Constanta and the “Victor Babes” University of Medicine and Pharmacy from Timisoara, Romania, adhering to established ethical guidelines. All participants were carefully told the purpose of the study, and written informed consent was obtained prior to the commencement of data collection. This procedure is in alignment with the principles set forth by the National Bioethics Commission of Romania that serves under the Declaration of Helsinki principles, ensuring the study’s adherence to the utmost ethical standards.

### 2.3. Creating the Romanian Version of the HALT (R-HALT)

The task of translating and cross-cultural adaptation was undertaken by a team of researchers who followed the existing guidelines [20]. Two academics from the dental field who were not familiar with the HALT questionnaire, with proficiency in English and with Romanian as their first language, independently translated the original HALT questionnaire from English to Romanian. A single Romanian version was established after comparing the two translations and resolving discrepancies by consensus. Following this, the new Romanian version was retro-translated to English by a professional translator. The same expert panel then compared the retro-translations to the original HALT questionnaire. Minimal variations concerning synonymous words were noted, and the retro-translation was forwarded to the original author, Dr. Victor Kizhner, for endorsement.

The Romanian version of the HALT was pre-tested on a sample of 20 individuals chosen by a convenience sampling method that enabled us to quickly gather data from a substantial number of young adults who were readily available and willing to participate. These individuals were not involved in the testing of the psychometric properties. Each participant was asked about any difficulties encountered while completing the preliminary tool, including understanding the questions, possible responses, or instructions. The team of authors subsequently reviewed the results and prepared the final version of the R-HALT. The cross-cultural adaptation process followed all necessary steps to ensure that the R-HALT attained conceptual and semantic equivalence across its 20 items.

### 2.4. Assessment of Psychometric Properties

To evaluate the psychometric properties of the Romanian HALT (R-HALT), the questionnaire was administered to a selection of volunteers attending dental clinics from the involved institutions. The sample size was determined following the existing recommendations that suggest a range of 2 to 20 individuals per item, with an absolute minimum of 100 to 250 individuals [21]. Given that the HALT comprises 20 items and considering a ratio of 5 individuals per item, a minimum of 100 individuals was set for the sample size.

Data were collected over a period from May to July 2023. Volunteers included university staff, professors, students, and patients receiving treatment at the dental clinics, all of whom were at least 20 years old. Exclusion criteria included individuals for whom Romanian was a second language, those with visual or hearing impairments, illiteracy, or individuals intoxicated by alcohol or drugs during the organoleptic evaluation. Young adults were considered between 16 and 26 years old. The organoleptic assessment of halitosis was carried out by a calibrated professional, instructing individuals to keep their mouths closed and breathe only through their noses for 3 min. Following this, they were asked to exhale through a paper tube 10 cm from the examiner’s nose. The intensity of oral malodor was then recorded using the 5-point scale outlined by the Rosenberg scale [22], in which 0 indicates no malodor, and 5 indicates severe malodor. A score of ≥2 was considered as a diagnosis of halitosis.

Participants received detailed instructions on how to complete the R-HALT questionnaire independently, in the absence of a researcher, to minimize potential bias. Questionnaires were disqualified if they were not fully completed or if participants could not be reached within a 7-to-10-day period from the initial application (for test–retest reliability) without modifying hygiene habits. Additionally, participants completed a demographic and socioeconomic form, as well as general and oral health questions.

Reliability testing was carried out solely with individuals diagnosed with halitosis. Convergent and discriminant validity testing, on the other hand, included all participants. To assess the stability of the R-HALT, it was administered twice within a 7-to-10-day interval. In the second administration, a dichotomous question was added: “Have you researched the topic in the interval between the first and second administration of the questionnaire?” This was carried out to mitigate any potential bias.

### 2.5. Statistical Analysis

All statistical analyses were performed using the SPSS v.26 for Windows (IBM Inc., Armonk, NY, USA), with a significance level of 5%. The R-HALT’s reliability was examined through its internal consistency (Cronbach’s alpha), with acceptable values being considered if higher than 0.70. The ICC correlation coefficient suggested a good correlation between 0.61 and 0.80 and an excellent correlation between 0.81 and 1.00. No item was excluded from the analysis. Considering that the R-HALT scores did not follow a normal distribution, Spearman’s correlation test was utilized to evaluate the convergent validity between R-HALT scores and the scores derived from the organoleptic method (considered the gold standard for halitosis diagnosis). For comparisons between groups, the Mann–Whitney and Kruskal–Wallis tests were employed.

The variables considered for analysis in the current study comprised the following: age, age range, gender, area of residence, body mass index, smoking status, alcohol consumption, the proportion of self-reported halitosis, the organoleptic test score, and the HALT survey results.

## 3. Results

### 3.1. Background Data

A total of 150 patients were recruited for the present study in the validation of the Romanian version of the Halitosis Associated Life-Quality Test (HALT) among young adults. The questionnaires were meticulously filled out, with the mean age of the sample being 23.6 ± 1.8 (16–26) years. The gender distribution consisted of 51% males (77 patients) and 49% females (73 patients). The age breakdown was as follows: 8% (12 patients) were between 16 and 18 years old, 25% (37 patients) were between 18 and 20 years old, 35% (52 patients) were between 20 and 22 years old, 22% (33 patients) were between 22 and 24 years old, and 10% (16 patients) were between 24 and 26 years old. Among these patients, 75% (112 patients) resided in urban areas, while 25% (38 patients) were from rural regions. In terms of BMI, 56% (84 patients) were in the normal weight range, 29% (44 patients) were overweight, and 15% (22 patients) were classified as obese.

As presented in Table 1, smoking was reported by 19% (29 patients), and alcohol consumption was noted in 52% (78 patients) of the sample. Self-reported halitosis was experienced by 21% (31 patients). The organoleptic test scores were distributed as follows: 41% (62 patients) were diagnosed with a score of 2; 29% (44 patients) displayed a score of 3; 21% (31 patients) had a score of 4; and 9% (13 patients) exhibited a score of 5.

### 3.2. Reliability of HALT Results

Table 2 presents the HALT results, illustrating various aspects of the questionnaire items’ validity and reliability. The mean scores of the items ranged from a low of 0.82 ± 0.94 (Q3) to a high of 3.23 ± 1.15 (Q11), reflecting the variability in the responses to different questions. The corrected item–total correlation was found to be generally moderate to strong across the items, with values ranging from 0.30 (Q2) to 0.90 (Q19). This indicated a substantial relationship between each item and the total score, signifying that the items were generally well-aligned with the underlying construct of the questionnaire.

The Cronbach’s alpha values if an item was deleted ranged from 0.93 to 0.96, with most values clustered around 0.94. This revealed that the internal consistency of the HALT scale was very robust, with none of the items substantially affecting the overall reliability if removed. It also demonstrated that the items were homogenous and measured the same underlying concept of halitosis-related quality of life. The highest corrected item–total correlation was observed in Q19 (0.90), whereas the lowest was in Q2 (0.30). The difference in these values highlighted the varying degrees of correlation with the total score, with Q19 having the most significant relationship with the underlying construct. Conversely, Q2 seemed to be the least related, although still moderately correlated.

Furthermore, the standard deviation values presented an insight into the dispersion of scores for each question. Items like Q19 (SD = 0.70) had less variability, indicating more consistency in the respondents’ answers, whereas others like Q1 (SD = 1.10) demonstrated greater dispersion in the scores. Test–retest reliability was also explored for a subset of 40 patients, with retesting conducted after a 2-week interval. The mean scores of the first and second measurements were 51.6 ± 12.9 and 47.3 ± 13.5, respectively, signifying stable measurements over time. An Intraclass Correlation Coefficient (ICC) value of 0.87 (95% CI = 0.70–0.99) was obtained, denoting excellent agreement between the measurements.

### 3.3. Analysis of Results

In assessing the HALT test’s construct validity, factor analysis was employed, revealing a KMO (Kaiser–Meyer–Olkin) test result of 0.80 and Bartlett’s test of sphericity value of 3559 (*p*-value < 0.001). As depicted in Table 3, the factor loadings for each item were all greater than 0.40, with values stretching from 0.53 to 0.94. Four main factors were extracted, resonating with the original English version, and together, they explained 85.18% of the total variance. These factors were defined as follows: Personal and Social Disabilities (27.32% variance, including items 14 through 20), Emotional Limitations (25.99% variance, covering items 4, 5, 10, 11, and 12), Functional Limitations (17.93% variance, involving items 6, 7, 8, 9, and 13), and Physical Limitations (13.94% variance, with items 1, 2, and 3).

**Figure 1:**
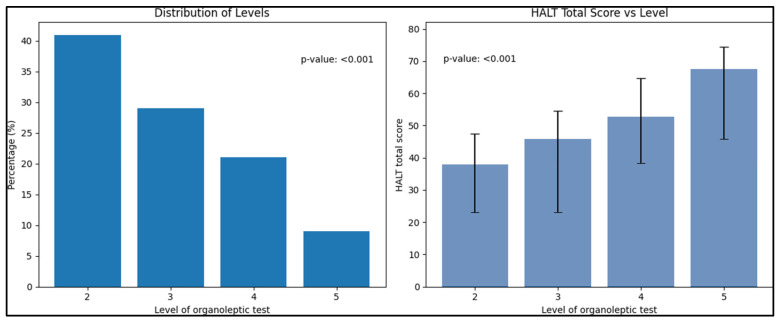
PRISMA flow diagram.

In addition, discriminative validity was explored through the Kruskal–Wallis test and Dunn’s post hoc test. Table 4 demonstrates the HALT scores based on varying degrees of oral malodor. The mean total scores (plus standard deviations) for the HALT were identified as 38.0 ± 9.4 for 2-level, 45.9 ± 11.8 for 3-level, 52.7 ± 12.0 for 4-level, and 67.5 ± 10.7 for 5-level oral malodor. Thus, a decrease in the quality of life of the respondents was identified by increasing the severity of halitosis, as objectively measured by the organoleptic test. Self-reported halitosis also showed statistical significance, with 58.4 ± 10.3 for “Yes” and 50.1 ± 1.08 for “No” responses. Both the organoleptic scale and self-reported halitosis showed *p*-values less than 0.001, highlighting a statistically significant difference. Dunn’s post hoc test confirmed remarkable differences in HALT scores across the groups, solidifying the statistical significance of these findings (*p* < 0.001), as described in Figure 1. The collective analysis reaffirms the HALT test’s validity and its alignment with the categorizations of oral malodor.

## 4. Discussion

### 4.1. Literature Analysis

The present study conducted a rigorous validation of the Romanian version of the Halitosis Associated Life-Quality Test (HALT) among young adults. The sample was comprised of 150 patients, reflecting a fair representation of both genders and a wide age range from 16 to 26 years. Our findings showed robust internal consistency and reliability in the HALT scores, with a high Cronbach’s alpha value (0.93 to 0.96) and an ICC value of 0.87. These findings are consistent with previous validations of the HALT questionnaire in other linguistic settings [16,23], reaffirming its effectiveness as a reliable instrument for assessing the quality of life related to halitosis.

The reliability of the HALT scale was examined through various statistical measures, demonstrating a very high internal consistency with Cronbach’s alpha values ranging from 0.93 to 0.96 [24]. Even the item with the least correlation to the overall score (Q2) still showed moderate alignment. The test–retest reliability also revealed excellent agreement, with an ICC value of 0.87. These results are consistent with the original validation of the English version of HALT [19] and underline the effectiveness of the Romanian version in maintaining the quality and integrity of the measurement. Moreover, the construct validity was evaluated through factor analysis, revealing four main factors mirroring the original English version. These factors, capturing more than 85% of the total variance, correspond to various dimensions of halitosis-related quality of life, including Physical, Functional, Emotional, and Personal and Social Disabilities. The alignment with the English version supports the generalizability of the HALT instrument across different linguistic contexts.

Further evidence of validity was found through discriminative analysis. The significant differences in HALT scores based on varying degrees of oral malodor and self-reported halitosis (*p*-value < 0.001) emphasize the instrument’s responsiveness to different levels of the condition. The results are aligned with previous studies, which have shown that patients’ self-perception of halitosis often correlates with clinical measurements [25,26]. This study also revealed correlations with lifestyle factors such as smoking and alcohol consumption. The association between these factors and oral malodor has been previously examined in the literature [27,28], reinforcing the complex nature of halitosis and its association with various lifestyle habits.

In accordance with our initial objectives, which encompassed the translation and validation of the HALT questionnaire, along with assessing the impact of halitosis on the quality of life, it is pivotal to emphasize the potential ramifications of halitosis observed in our study. As illustrated by the results, the discriminative analysis clearly indicates a progressive increase in HALT total scores with higher levels of the organoleptic test and a significant difference in scores between individuals who self-reported halitosis compared to those who did not. This trend showcases a direct correlation between the perceived severity of halitosis and the deteriorating quality of life. Particularly notable is the substantial rise in the HALT score at the highest level of the organoleptic test (67.5 ± 10.7), which signals a pronounced negative impact on the quality of life for individuals in this category. This result underscores the necessity to address halitosis not merely as a physiological condition but as a significant determinant influencing the quality of life. Future studies may delve deeper into investigating the multifaceted impact of halitosis on the quality of life, encompassing psychological, social, and emotional dimensions, thereby furnishing a comprehensive perspective on the gravity of the issue.

Our sample had an almost even gender distribution and included the age groups between 16 and 26 years old. The age-related variations in halitosis have been explored in other studies [29], but our research further emphasizes the need to understand halitosis in younger populations. The observation that 75% of the sample resided in urban areas and 25% in rural regions could provide insights into potential environmental and cultural differences in oral health practices. Previous studies have shown disparities in oral health between urban and rural populations, although in different settings and backgrounds compared to Romania [30]. The classification of participants into normal weight, overweight, and obese categories might suggest a potential link between body weight and oral health. This is consistent with previous findings that have noted correlations between obesity and oral health problems [31].

The method of halitosis measurement used in our study was the clinical evaluation, typically an objective examination known as the organoleptic method, where the odors from patients are smelled directly. The advantages of this approach include its low cost, lack of equipment requirements, and the ability to detect a wide range of odors. However, its downsides encompass extreme subjectivity, lack of quantification, nose saturation, and low reproducibility, even though it is considered the gold standard in halitosis diagnosis [4]. In our research, the organoleptic method was employed to assess the tool’s convergent validity with the R-HALT test, which gauges the negative impact of halitosis on oral health-related quality of life. The results affirmed that individuals with higher R-HALT scores experienced more severe halitosis. Additionally, the average total score was notably higher for individuals who self-reported having halitosis, showcasing this difference as evidence of discriminant validity. These findings have implications in comparison with more subjective methods of measurement, such as the HALT survey, emphasizing both the benefits and limitations of using the organoleptic method. Nevertheless, various devices have been identified that detect the volatile compounds causing malodor, yet the organoleptic test retains its status as the gold standard due to machines’ limitations in detecting only volatile sulphury compounds, unlike other odor measurement tools that can recognize complex substance mixtures and provide false results [32].

Lastly, it is important to acknowledge that the percentage of halitophobic patients is notably high, varying by country from 20% to 50% [33]. Two-thirds of these patients are women, and this phenomenon is partly attributed to increased self-consciousness and advertising of oral hygiene products [34]. Other studies emphasize the discrepancy between self-reported halitosis and its objective presence; however, a correlation was found in patients with slight or moderate halitosis between their self-assessment and the organoleptic examination [35]. This information offers important insights into the complexities of diagnosing and understanding halitosis and may have significant implications for our study.

### 4.2. Study Limitations

One of the main limitations of this study resides in the method of sampling by utilizing a convenience sampling method for pre-testing the Romanian version of HALT (R-HALT) that might not fully represent the broader population and could introduce a bias, affecting the generalizability of the results. Moreover, while this study ensures cross-cultural adaptation and demonstrates robust psychometric properties, minimal variations concerning synonymous words were noted in the translation, which may have affected the semantic equivalence. The exclusion criteria, including individuals for whom Romanian was a second language or had visual or hearing impairments, may further limit the applicability of the findings. This study’s sample size, while determined according to existing recommendations, could be considered relatively small, potentially affecting the statistical power. Additionally, the age range of this study was confined to young adults, limiting the insights into how HALT applies to other age demographics. The dichotomous question added in the second administration to mitigate potential bias might not be entirely effective in eliminating respondents’ influence, having researched the topic in the interval between the two administrations. Lastly, the results were confined to a specific cultural and linguistic context, which may hinder the ability to extrapolate the findings to other cultural or linguistic groups.

## 5. Conclusions

The validation of the Romanian version of the Halitosis Associated Life-Quality Test (HALT) in this study confirms its robust reliability and validity in assessing the impact of halitosis on young adults, highlighting a marked influence of halitosis on quality of life. The notable correlation between organoleptic test scores and HALT scores accentuates the necessity for comprehensive research focusing on the broader impacts of halitosis. The tool’s internal consistency, agreement over time, and strong correlation with oral malodor categorizations contribute to its credibility. Furthermore, the factor analysis aligns well with the original English version, supporting its applicability in the local context. The statistically significant differences detected in the discriminative analysis reinforce the HALT test’s potential to differentiate levels of oral malodor. Future studies could explore the application of the HALT questionnaire across different age groups and settings in Romania. Moreover, healthcare providers may consider using this validated instrument to inform patient care decisions and interventions related to halitosis, contributing to enhanced individual well-being and public health strategies.

## Figures and Tables

**Table 1:** Background analysis.

Variables	Number	%
Age (mean ± SD)	23.6 ± 1.8	–
Age range		
16–18 years	12	8%
18–20 years	37	25%
20–22 years	52	35%
22–24 years	33	22%
24–26 years	16	10%
Gender		
Male	77	51%
Female	73	49%
Area of residence		
Urban	112	75%
Rural	38	25%
BMI		
Overweight	44	29%
Obese	22	15%
Normal	84	56%
Smoking (yes, %)	29	19%
Alcohol consumption (yes, %)	78	52%
Self-reported halitosis (yes, %)	31	21%
Organoleptic test score		
2	62	41%
3	44	29%
4	31	21%
5	13	9%

BMI—Body Mass Index; SD—Standard Deviation.

**Table 2:** HALT results.

Item	Mean ± SD	Corrected Item– Total Correlation	Cronbach’s Alpha If Item Deleted
Q1	2.00 ± 1.10	0.49	0.95
Q2	1.05 ± 0.83	0.30	0.94
Q3	0.82 ± 0.94	0.42	0.94
Q4	3.10 ± 1.25	0.67	0.94
Q5	3.15 ± 1.18	0.71	0.95
Q6	2.35 ± 0.95	0.70	0.95
Q7	1.58 ± 0.92	0.75	0.93
Q8	2.45 ± 0.85	0.52	0.93
Q9	1.38 ± 0.79	0.60	0.93
Q10	3.18 ± 1.20	0.69	0.93
Q11	3.23 ± 1.15	0.74	0.94
Q12	2.92 ± 0.92	0.82	0.95
Q13	2.95 ± 1.00	0.77	0.95
Q14	3.07 ± 1.05	0.80	0.96
Q15	2.05 ± 0.75	0.83	0.94
Q16	2.75 ± 1.08	0.85	0.94
Q17	2.43 ± 0.90	0.71	0.94
Q18	2.78 ± 0.88	0.59	0.94
Q19	3.12 ± 0.70	0.90	0.94
Q20	3.22 ± 0.72	0.82	0.93

SD—Standard Deviation.

**Table 3:** Factor analysis.

Item	Factor 1	Factor 2	Factor 3	Factor 4
Q1	0.91	0.33	0.06	0.5
Q2	0.88	0.32	0.25	0.19
Q3	0.81	0.41	0.23	0.30
Q4	0.80	0.44	0.11	0.23
Q5	0.78	0.21	0.40	0.21
Q6	0.76	0.09	0.48	0.16
Q7	0.63	0.57	0.10	0.36
Q8	0.15	0.93	0.17	0.09
Q9	0.24	0.92	0.08	0.12
Q10	0.23	0.9	0.18	0.10
Q11	0.29	0.88	0.17	−0.14
Q12	0.31	0.58	0.53	0.27
Q13	0.05	0.11	0.82	0.23
Q14	0.34	0.45	0.73	−0.08
Q15	0.23	0.34	0.65	0.33
Q16	0.33	0.03	0.68	0.44
Q17	0.38	0.33	0.52	0.42
Q18	−0.10	0.02	0.31	0.85
Q19	0.51	−0.08	−0.06	0.69
Q20	0.18	−0.02	0.40	0.76
Eigenvalue	5.40	5.11	3.55	2.89
Percent variance	27.2%	25.5%	17.8%	14.0%

**Table 4:** Discriminative analysis.

Variable	n (%)	HALT Total Score (Mean ± SD)	*p*-Value
Level of organoleptic test			<0.001
2	62 (41%)	38.0 ± 9.4	
3	44 (29%)	45.9 ± 11.8	
4	31 (21%)	52.7 ± 12.0	
5	13 (9%)	67.5 ± 10.7	
Self-reported halitosis			<0.001
Yes	31 (21%)	58.4 ± 10.3	
No	119 (79%)	50.1 ± 1.08	

SD—Standard Deviation.

## Data Availability

Data are available upon request.
